# Catalase as a Molecular Target for Male Infertility Diagnosis and Monitoring: An Overview

**DOI:** 10.3390/antiox9010078

**Published:** 2020-01-16

**Authors:** Nuria Rubio-Riquelme, Natalia Huerta-Retamal, María José Gómez-Torres, Rosa María Martínez-Espinosa

**Affiliations:** 1 Biotechnology Department, Faculty of Sciences, University of Alicante, Carretera San Vicente del Raspeig s/n—03690 San Vicente del Raspeig, 03690 Alicante, Spain; nuriarubio.152@gmail.com (N.R.-R.); natalia.huerta@ua.es (N.H.-R.); mjose.gomez@ua.es (M.J.G.-T.); 2 Agrochemistry and Biochemistry Department, Biochemistry and Molecular Biology Division, Faculty of Sciences, University of Alicante, Carretera San Vicente del Raspeig s/n—03690 San Vicente del Raspeig, 03690 Alicante, Spain

**Keywords:** catalase, reactive oxygen species (ROS), oxidative stress, male fertility

## Abstract

Catalase (CAT) stands out as one of the most efficient natural enzymes when catalysing the split of H_2_O_2_ into H_2_O and O_2_; H_2_O_2_ is one of the reactive oxygen species (ROS) involved in oxidative stress, a process closely related to aging and several health disorders or diseases like male infertility. Some studies have correlated H_2_O_2_ with male infertility and catalase with fertility restoration. However, the number of studies conducted with human beings remains scarce. Considering the use of CAT as a molecular target for biochemical analysis, this review summarises the current knowledge on how CAT influences human beings’ male fertility. Thus, three different databases were consulted—Scopus, PubMed and WOS—using single keywords and combinations thereof. A total of 40,823 articles were identified. Adopting inclusion and exclusion criteria, a final database of 197 articles served to conduct this work. It follows from this analysis that CAT could play an important role in male fertility and could become a good target for male infertility diagnosis and monitoring. However, that potential role of CAT as a tool in diagnosis must be confirmed by clinical trials. Finally, guidelines are suggested to reinforce the use of CAT in daily clinical tests for male fertility diagnosis and monitoring.

## 1. Introduction

Infertility is described as a disease of the reproductive system characterised by the failure to establish a clinical pregnancy after 12 months of regular, unprotected sexual intercourse or due to an impairment of someone’s capacity to reproduce, either as an individual or with their partner [1]. Based on the latest definition offered by the World Health Organisation (WHO), it is likewise a disease that generates disability as a function impairment [2]. Over 186 million people suffer from infertility worldwide, most of them residents in developing countries [3]. Approximately 15% of couples cannot conceive a child after the 12 months established by WHO [4], the male factor alone being responsible for 20–30% of infertility cases and significantly affecting 50% of all cases [5].

These high rates stress the key importance of appropriately and concurrently managing male fertility. The evaluation of infertile males aims to identify correctable causes of infertility that can guide an infertile couple’s therapeutic decision making [6]. Other significant medical problems related to infertility can also be detected, though, such as Multiple sclerosis, Pituitary adenoma, Cystic fibrosis, Diabetes, Hypopituitarism, Klinefelter syndrome, Prostate cancer, Spinal cord tumours, Testis cancer, Thyroid disease or Urinary tract infection [7,8]. Fortunately, significant progress has been made during the past 50 years in understanding male infertility, which, in turn, has served to promote new approaches which materialise in novel diagnostic and therapeutic strategies meant to help this cohort of infertile patients [6].

Along with a thorough history and physical examination, semen analysis has long been the pillar for male infertility evaluation and management. Although abnormal semen analysis correlates to the decrease of natural conception, it cannot draw a distinction between fertile and sterile patients (except for azoospermia) [9]. This methodology fails to answer the more relevant clinical question of which specific semen parameters and cut-offs can distinguish male fertility from subfertility. Efforts to identify those parameters have instead found that while semen parameters are associated with fecundity, neither sperm concentration, morphology, nor motility could be considered diagnostic of infertility either alone or in combination [9,10]. Male infertility evaluation is usually based on a simple seminogram—a test which is currently considered of little use due to the uncertainty that it still presents [11]. Consequently, seminogram needs to be complemented by a comprehensive medical history on the basis of a physical examination, and significant endocrine, genetic, and other biochemical investigations [6,12]. Furthermore, the seminogram must continue evolving so that new parameters can be studied to provide more information and reduce the current uncertainty, as well as to diagnose male infertility and its causes more accurately.

The etiological factors for male infertility are highly diverse, including physiological and environmental factors, genetic, immunological and endocrine disorders, and infections or obstructive lesions in the male reproductive tract. Moreover, the etiology remains unknown and the infertility is classified as idiopathic in more than 50% of male infertility cases [4,12,13,14]. A new but important cause has emerged next to the conventional origins of male infertility: oxidative stress (OS)—described as the main etiological factor causing sperm DNA damage [15,16,17].

Among the biochemical parameters of disease diagnosis soundness, enzymes arise as suitable biomolecules not only for diagnosis but also for monitoring purposes. Considering the links established between oxidative processes and infertility in human beings, enzymes related to these processes seem appropriate candidates for studies on human fertility. This review consequently summarises the main current knowledge about the role of the catalase (CAT) enzyme when it comes to male infertility. CAT highly efficiently catalyses the decomposition of H_2_O_2_ into H_2_O and O_2_. H_2_O_2_ additionally stands out as one of the reactive oxygen species (ROS) related to oxidative stress, a process closely related to aging and several health disorders or diseases like male infertility. Few studies have hitherto correlated H_2_O_2_ with male infertility and CAT with the restoration of fertility. However, the number of studies conducted with human beings remains scarce and not many of them are based on clinical trials. This analysis can thus help to better understand what has been achieved to date, highlighting the most important open questions about this field of knowledge that will require deeper research in the coming years.

## 2. Materials and Methods 

### 2.1. Search Strategy and Information Processing

Firstly, searches of generic character were carried out using the “Google scholar” portal (https://scholar.google.es/) which led us not only to identify several key concepts but also to filter the keywords that had to be subsequently utilised in a more exhaustive search across scientific databases. The final list of keywords stemmed from the “MeSH database” (Medical Subject Heading) and NLM (The National Library of Medicine).

Secondly, a complete search carried out on platforms connected to databases related to the topic under study made it possible both to conduct a more precise bibliographic and bibliometric analysis and to know the bibliographic load indexed in each one of the databases used. The selected databases were PubMed, Scopus and Web of Science (WOS). 

Three inclusion criteria served to refine the search and to select the documents: (i) a 25-year study period (1993–2018); (ii) type of articles: reviews and clinical trials from primary sources and indexed journals; and (iii) studies published in English. Furthermore, only studies framed within the area of reproductive biology research ended up being part of the database prepared for this study.

Finally, advanced search took place using the WOS database for the purpose of identifying the documents of interest. The option “Search all databases” was selected before starting the research. The field tags “topic” and “title” were chosen, the information retrieval system “Boolean” serving to identify the studies of interest for this review by means of the following keywords: “catalase”; “oxidative stress”; “male fertility”; “catalase & oxidative stress”; “catalase & male fertility”; “oxidative stress & male fertility.” Hydrogen peroxide was also used as keyword at the very beginning in this research. Considering that hydrogen peroxide is related to thousands of different metabolic processes, the information obtained was quite dispersed and not relevant for the aim of this research. Consequently, this keyword was not included. Finally, the RefStudies program for bibliographic data administration allowed us to avoid duplicating the papers obtained in each single search.

### 2.2. Selection of Relevant Studies and Data Analysis

The initial 40,823 articles identified went down to 1667 after applying the inclusion criteria. The searches were made independently (one for each author), the database for this review eventually being confined to the 197 papers which belonged to the area of reproductive biology. Seeking to identify the relevant studies for the present research, both the aforementioned inclusion criteria and the following exclusion criteria enabled us to build the final database: (i) studies which did not have male fertility as their main topic; (ii) studies based on non-specific systematic review; and (iii) non-human clinical trials were discarded. Out of the 109 articles which remained after the application of these criteria, only 18 studied the relationship between catalase activity and male fertility; hence our decision to examine them more closely (see Tables S1 and S2). Each of the final database articles was analysed by the four authors with backgrounds in biology, reproductive medicine, and biochemistry. The classic scheme proposed by Vilanova (based on PRISMA (http://prisma-statement.org/Protocols/)) served to ensure selection criterion quality [18]. The following questions were thus suggested to evaluate each of the identified studies: is it related to the main research objectives of this review? Is the methodology clear and objective? Is the study 100% reproducible? Is the sample size coherent? Is the sample well defined? Is the sample representative? Is the hypothesis clearly stated?

Articles summarising results from studies based on clinical trials (8 of the 18 articles selected) received special attention (Table 5), heterogeneity of the clinical trials design being the most important limitation faced in this research. None of these articles provided suitable data to calculate heterogeneity statistics.

## 3. Results and Discussion

### 3.1. Compilation of Relevant Bibliographic Sources

After a general/an overall search through Google scholar, the major search engines PubMed, Scopus and Web of Science helped us to undertake more detailed and accurate research. An initial search based on the keywords “catalase”; “male fertility” and “oxidative stress” was conducted in each of these databases. Those key words were used either alone or combined in pairs, using one of the established inclusion criteria at a time to know which one was the most limiting (the type of document selected turned out to be the most limiting criterion in every case).

A joint database in RefStudies was subsequently elaborated to include the articles obtained from each single search using the engines mentioned above. In the case of Scopus, its characteristics prevented that search engine from exporting more than 2000 articles—which made it impossible to bring all the articles obtained together in a single bibliographical database. Furthermore, although it became clear from this search that Scopus reported a greater number of articles than WOS, the type of document “clinical trial” could not be included during the search. The number of original papers identified through WOS significantly exceeded those identified by PubMed, and more importantly, WOS was the only database among the three initially selected where articles can be defined according to the research area.

Afterwards, a new search method was proposed which used combinations of the keywords in the different databases. The information retrieval system “Boolean” permitted to identify the studies of interest for this review, with “catalase & oxidative stress”; “catalase & male fertility” and “oxidative stress & male fertility” as keywords. The advanced search form additionally ensured that the articles shown met the inclusion criteria specified above. However, despite the small number of articles, non-specific and unrelated results were detected for the main subject matter of this review (mainly in the combination of the keywords “catalase” & “oxidative stress”).

This led to the selection of a single database for bibliometric analysis based on the number of publications reported by each of them and the chances to select the required research area as an inclusion criterion. It became visible through this new search that WOS was the best database to identify and select the papers of interest for this study, because a larger number of publications can be identified and the possibility exists to select the research area as an inclusion criterion, thus avoiding unspecific results (Table 1).

A total of 40,823 studies were identified after combining the three keywords by pairs in WOS. Only 1667 of them met the inclusion criteria and a much smaller number, 208, also belonged to the area of reproductive biology research. The analysis of these 208 articles revealed that 156 (75%) studies contained systematic reviews and 52 (25%) described clinical cases. After including them in the database created using RefStudies, duplicated articles were excluded, which left us with 197 studies—0.5% of the initial total—which then came to form the database to be ultimately analysed (Table 2). The remaining 197 studies were studied individually in order to discard those which met any of the exclusion criteria specified in the materials and method section of this paper. A total of 88 articles were accordingly discarded because at least one of the exclusion criteria was applicable to them (Table 3).

**Figure 1:**
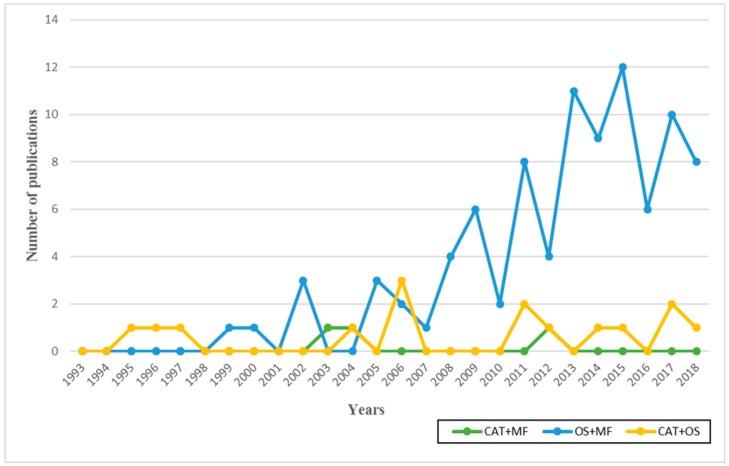
Number of publications per year (January 1993–December 2018) and by keyword. Database created after applying the inclusion and exclusion criteria.

The analysis of the 109 documents finally included in this study reveals that some of the earliest publications devoted to catalase and oxidative stress, as well as to the effect of oxidative stress on male fertility, date back to the 1990s—with a rate of one publication per year throughout that decade. However, the first studies to relate male fertility and catalase did not appear until 2003 (Figure 1).

The number of publications on oxidative stress (OS) and male fertility (MF) is strikingly low compared to other biomedical research fields. Nevertheless, the number of publications dealing with this topic over the years shows a clear tendency to gradual increase, the highest score being recorded in 2015 with a total of 12 publications identified using the combination “OS + MF.” The combinations of “CAT + OS” and “CAT + MF” have revealed low and stable publication numbers throughout the period analysed (last 25 years). These observations probably have to do with the fact that the study of oxidative stress in relation to male fertility is a more generic field than the other two mentioned above. After all, it covers many pathologies and conditions that are likely to affect OS levels by impairing male fertility; instead, specific studies about the CAT among the large number of antioxidants are much less often found. Finally, the smaller number of publications on the study of CAT in relation to male fertility—only 3 in the last 25 years—deserves to be highlighted. This difference may be due to the limited study of CAT in humans—at least related to male fertility—despite the unquestionable relationship between both aspects.

It is additionally worth mentioning that the study of the role played by CAT on oxidative stress and male fertility raises worldwide interest, as shown by the fact that the publications identified came from different countries around the world and were attributed to a wide variety of institutions and authors (Table 4). 

### 3.2. Bibliographical Analysis

#### 3.2.1. ROS and Male Fertility

The oxidative stress state (OSS) takes place when an imbalance exists between ROS production and antioxidant activity. Its evaluation can play a fundamental role in the control of sperm damage and male fertilising capacity, insofar as studies have revealed that almost 40% of infertile males display abnormally increased ROS levels [19]. Human spermatozoa are known to be extremely sensitive to ROS, which can be generated both by endogenous physiological processes—e.g., mitochondrial respiration and seminal leukocytes [15,20,21]—and by a number of environmental factors which largely depend on the individual’s lifestyle and ranging from drugs, pollution, toxins, tobacco and radiation, to diet itself [19,22]. “Normal physiological ROS concentrations” are not harmful, though. In fact, they constitute a key requirement for sperm to acquire fertilising capacity and facilitate the adequate development of essential processes such as capacitation, hyperactivation, acrosome reaction and the fusion with the oocyte [23,24]. 

Antioxidants work by halting the oxidative chain reaction, removing, taking up or decreasing ROS formation [25], and thus avoid the potential imbalance between oxidants and antioxidants that would cause oxidative stress. Aerobic cells have evolved to become specific enzymatic ROS scavengers, including superoxide dismutase (SOD, EC 1.15.1.1), catalase (CAT, EC 1.11.1.6), and glutathione peroxidases (GPX, EC 1.11.1.9), which work closely together [26]. 

The imbalance between ROS production and antioxidants proves detrimental to the functional and structural integrity of highly differentiated cells like spermatozoa [26,27,28,29,30]. When this situation arises, significant oxidative damage occurs in numerous cell organelles through a degradation of lipids, proteins, DNA, and carbohydrates, which ultimately results in cell death [15]. The damage caused by this stress seemingly constitutes a significant factor in 30–80% of all male infertility cases [21,31,32]. Sperm is very prone to oxidative damage due to the large number of mitochondria that it possesses and because of its unique structural composition. Since sperm has a small cytoplasmic fraction (where most cells contain greater amounts of antioxidants), cells are more susceptible to damage [15,33,34]. ROS mainly prove harmful to the plasma membrane of sperm as a result of the high polyunsaturated fatty acid content which characterises such membranes [35,36]. Those fatty acids are in turn more susceptible to lipid peroxidation than other non-germinal cells [37], which causes anomalies in sperm cells, including membrane damage and decreased mobility [37,38]. Only one of the studies included in this review stated that low levels of oxidative species could negatively affect DNA structure and stability [30], whilst the rest of the studies demonstrated that low levels of ROS (usually considered “normal physiological ROS concentrations”) are required for several processes related to human fertility [23,24]. Thus, only at high oxidative stress levels, sperm chromatin begins to fragment, thus decreasing DNA integrity and fertilising capacity [30,39]. 

Different research groups have recently coincided in identifying hydrogen peroxide (H_2_O_2_) as the most cytotoxic oxygen metabolite within this context [37,40,41,42]. In cases in which the loss of sperm functions is mainly due to H_2_O_2_, catalase, which selectively degrades this ROS, is the only scavenger that grants complete protection to spermatozoa [38,43,44,45], additionally helping to prevent the loss of sperm motility in such circumstances [37,40,41,42]. This antioxidant capacity was demonstrated by observing that sperm lost motility in an oxygenated medium, which could later be recovered through catalase enzymatic activity [30,46]. Evidence was subsequently found of sperm producing reactive oxygen species, more specifically H_2_O_2_ [47]. Thus, H_2_O_2_ could play a dual role, either being necessary for sperm metabolism at certain concentrations or behaving as a cytotoxic compound above these concentrations [23].

#### 3.2.2. Catalase and Male Fertility

Catalase (EC 1.11.1.6)—a tetrameric protein (500 amino acids per chain)—contains four heme groups which react with hydrogen peroxide [48]. This enzyme catalyses the conversion of 2 H_2_O_2_ molecules into 2 molecules of water and molecular oxygen, respectively [49] (Equation (1)). The reaction catalysed by catalase (CAT) plays a role not only in the maintenance of normal ROS levels but also in the protection of spermatozoa against potentially toxic ROS levels [50]. It becomes the key enzyme in reproductive processes and shows one of the highest catalytic efficiencies described so far: a molecule of catalase can transform millions of hydrogen peroxide molecules into water and oxygen per second [51]. This reaction consequently prevents the conversion of H_2_O_2_ into hydroxyl radicals and other more toxic ROS [52]. This enzyme likewise shows a high degree of effectiveness with augmented oxidative stress and has been found not only in human spermatozoa but also in seminal plasma of fertile as well as infertile individuals [53,54].

(1) $$2H_{2}O_{2}\to 2H_{2}O+O_{2}$$

Previous research has proven that seminal plasma superoxide dismutase (SOD), catalase (CAT), glutathione peroxidase (GPx), and sulfhydryl group levels are significantly lower in infertile patients than in those belonging to control groups, which clearly suggests their direct connection with male fertility [55]. Added to this, a number of authors have stated differences in the seminal catalase activity of asthenozoospermic and oligo-asthenozoospermic specimens with hyperviscosity [56], although the role of catalase in the ejaculate in relation to male fertility had been insufficiently studied at the time of writing this review.

### 3.3. Current Evidence Sustaining the Relationship between Catalase and Male Fertility

Despite the remarkable progress made in assisted reproduction, many men with normal parameters in standard seminal analyses remain infertile. This suggests that a routine semen analysis does not necessarily suffice to provide complete diagnostic information. Among the various possible causes of idiopathic infertility, different research groups have mentioned a state of oxidative stress as a potential cause [15,23,25]. Sharma and Agarwal [57] reached the conclusion that ROS intrinsic reactivity, particularly that of H_2_O_2_ and the superoxide anion, seems to be a major reason for defective sperm function in male infertility cases despite the antioxidant properties of seminal plasma. To which they added that determining the male infertility etiology is of paramount importance, insofar as it can help to develop effective therapies to overcome excessive ROS generation [57]. This conclusion was also drawn by other groups, whose investigations equally highlighted the importance of developing an effective oxidative stress assessment system for the study of male fertility [20,58]. This would not only allow us to analyse the fertility “status” but also to identify the sub-groups of patients that respond or fail to respond to different therapeutic strategies [57]. A multi-faceted therapeutic approach to male fertility improvement requires identifying harmful environmental and occupational risk factors, while simultaneously correcting underlying nutritional imbalances to encourage optimal production and function [58]. The next sections provide more specific details about each one of the aspects already identified as key factors in relation to catalase and male fertility.

#### 3.3.1. Antioxidant Therapies 

Therapies with antioxidant compounds or dietary supplements have been widely evaluated as a treatment option for male infertility cases, since nutritional therapies have been shown to improve sperm counts and sperm motility, to quote but two sperm parameters [59,60,61]. Several studies set themselves the objective of examining the effects caused by different types of antioxidant therapies on semen quality in infertile males through a measurement of CAT activity on seminal plasma through exogenous H_2_O_2_ degeneration (this can be easily measured by means of spectrophotometry). These studies revealed that catalase activity increased after the administration of antioxidant treatments compared to control samples without antioxidant supplementation [60,61,62]. 

A few studies have evaluated the role of vitamins and antioxidants in male infertility, mostly suggesting a beneficial effect of the antioxidant therapy in male infertility treatments [60,62]. Tartibian and Maleki found in a small, double-blind, randomised placebo-controlled trial that supplementation with honey attenuated the possible aggravating effects of high-intensity training on spermatogenesis, CAT activity increasing in seminal plasma [62]. In turn, a larger double-blind randomised placebo-controlled trial carried out by Safarinejad [60] reported a significant correlation between the increased CAT activity in seminal plasma and an improvement in the different/various seminal quality parameters studied after the treatment with pentoxifylline (PTX). Furthermore, 3 pre-treatment seminograms were carried out—instead of only/one—seeking to minimise the effect of possible spontaneous variations. Similarly, a drop in the values obtained for catalase and seminal quality became clear during the post-treatment period [60]. 

Coenzyme Q_10_ (CoQ_10_)—a mitochondrial respiratory chain component—plays a key role in energetic metabolism. Furthermore, it is an important liposoluble chain-breaking antioxidant associated with biological membranes and lipoproteins [58]. In contrast to the positive studies mentioned above, Nadjarzadeh and co-workers reported no beneficial effects on sperm quality derived from the administration of CoQ_10_ in a small, placebo-controlled trial, despite obtaining a significant increase in catalase activity after the treatment [61]. 

Even though antioxidant therapies managed to report benefits, administering certain chemical compounds sometimes gives rise to side effects [60]. An example can be found in many plant-derived substances—collectively termed “phytonutrients” or “phytochemicals”—which are arousing interest because of their antioxidant activity [58]. For instance, the Indian herb *Withania somnifera* has been commonly used to treat male infertility. Its use extends to the treatment of erectile dysfunction, oligozoospermia, reproductive endocrinological problems and many other male reproductive health problems [63]. Sengupta and co-authors referred to several studies exploring the effects of *Withania somnifera* on the quality of semen in infertile males, all of which showed that the amount of LPO (lipid peroxidation) was found to decrease after administering this herb. Such an effect most probably has to do with a synergistic relationship between the increase of two enzymatic activities (SOD and CAT) combined with the innate antioxidant activity of *Withania somnifera* [63].

Finally, despite the evidence provided by in vitro studies according to which antioxidant supplements positively influence the protection of sperm DNA against exogenous oxidants, the effect of these antioxidants in protecting sperm from endogenous ROS, gentle sperm processing and cryopreservation remains unclear [59].

#### 3.3.2. Pathology of the Male Genital Tract and Genetics Related to CAT and Male Fertility

Antioxidant genes—which include genes encoding CAT—play relevant roles in spermatogenesis and normal sperm function [59]. According to Yu and Huang, genetic variations in major antioxidant genes might alter a male’s proneness to infertility and defective spermatogenesis. Nevertheless, the study about most of the modifications affecting antioxidant genes apart from CAT has been mostly confined to animal models [64].

Diseases related to the male reproductive system may similarly turn out to be the cause behind decreased antioxidant enzyme activity, which ultimately leads to an OSS. The prostate gland plays an important role in male reproduction. Its inflammation (prostatitis) stands out as a common health problem that affects many young and middle-aged men. Despite being regarded as a correctable cause of male infertility, both the pathophysiology and the appropriate treatment options for prostatitis in male infertility remain unclear [65]. Zhou and co-authors observed a significantly decreased activity of CAT—among other antioxidants—in patients with chronic bacterial prostatitis as opposed to healthy subjects (randomised case-control study) [66]. Even though ROS are usually scavenged by antioxidants, lower antioxidant concentrations may leave a surplus of ROS that results in higher oxidative stress levels, followed by oxidative damage in more prolonged cases [67]. Male reproductive tract bacterial infections, including chronic bacterial prostatitis, negatively affect sperm quality as well. In contrast, other studies fail to demonstrate any differences in standard sperm parameters like semen concentration, sperm motility, and sperm morphology when prostatitis patients were compared to those belonging to control groups [65]. 

#### 3.3.3. Lifestyle Factors: Harmful vs. Beneficial Factors

The examination of the crucial role played by modifiable lifestyle factors in the development of male infertility has been generating more and more interest lately. Studying factors such as psychological stress, nutrition, physical activity, alcohol intake, toxic pollutants and mobile phone use allows us to ascertain their possible effects on semen quality [68,69]. Although the imbalance between ROS and antioxidant generation is likely to occur naturally as a result of internal factors, a harmful lifestyle can largely favour oxidative stress development. Different substances such as tobacco and alcohol have received extensive attention in the context of male fertility. Their consumption triggers the appearance of an OSS that eventually affects seminal quality and causes reproductive hormone system dysfunction and impaired spermatogenesis, as well as sperm maturation and spermatozoa function [69,70,71]. Similarly, other harmful habits such as mobile phone overuse have been associated with deterioration in male fertility—owing to the emission of radiofrequency electromagnetic waves (RF-EMW) towards nearby base stations or relay antennas (the human body acts as an antenna absorbing radiation). Some authors try to explain the deterioration of male reproductive potential due to the abusive use of cell (mobile) phones [72,73]. Chronic exposure to RF-EMW decreases CAT, SOD and GPx activity, thus reducing the total antioxidant capacity [73]. 

The previously mentioned harmful lifestyles may affect the ROS/antioxidant balance in semen either by increasing ROS or by reducing antioxidant capacity. The suggestion from a pathophysiological point of view is that the oxidative stress caused by this imbalance directly correlates associated with sperm DNA damage and, subsequently, apoptosis; and these phenomena work as continuous molecular mechanisms potentially leading to impaired male fertility [73,74]. 

According to Dai and co-workers, antioxidants such as CAT are reduced in smokers. A tendency exists, however, to observe a significant relationship between tobacco consumption and male infertility. The controversy which still arises when comparing mainly relates to the lack of enough insights into the underlying mechanisms that sustain the toxicological effects observed [71]. Regarding alcohol, Maneesh et al. observed a significant relationship in their case-control study between a chronic excessive alcohol consumption and an increased OS, which, in turn, causes lipid peroxidation to grow largely. A significant decrease in CAT activity additionally became clear, probably resulting from the lower availability of nicotinic acid dinucleotide phosphate-reduced (NADPH) [70]. 

The same as an unhealthy lifestyle can impair reproductive capacity, the adoption of healthy habits is bound to improve it. Thus, oxidative stress can be defeated by adopting healthy habits such as playing sports or following a healthy diet. Emerging data suggest that regular exercise improves male reproductive function markers, including semen quality parameters and oxidative stress levels, in both fertile and infertile populations [75,76,77]. According to Maleki and Tartibianthe, the effects of a physically active lifestyle on male reproduction can be primarily attributed to its anti-inflammatory and antioxidant properties [76]. A randomised controlled trial served to study three different physical exercise intensities regarding OS markers, seminal quality and DNA integrity. Seminal quality significantly improved after 24 weeks of physical activity, as well as sperm DNA integrity, accompanied by an increased CAT activity that could be correlated with these seminal parameters. It additionally became clear that the higher CAT activity was the only parameter that remained high after 30 days of training [75]. Even though some studies suggest that doing high-intensity exercise can result in a higher OS [62], Maleki et al. reported an improvement in OS and seminal quality markers in every intensity range studied, as opposed to the results obtained for subjects who lead a sedentary life. Nevertheless, continuous moderate-intensity training turned out to be more beneficial to improve male reproductive function markers than high-intensity exercise. These observations suggest that parameters such as the intensity, the duration and type of exercise training should be taken into consideration when investigating reproductive responses to training in men [75]. 

A similar randomised controlled trial examined the ability to improve reproductive function in relation to the combined practice of aerobic and anaerobic exercises. Both moderate aerobic exercise training and resistance training were reported to decrease lipid peroxidation levels and to increase protection against oxidative stress [76]. Unlike Maleki et al. [75], Maleki and Tartibian [76] took as the starting point for their study subjects with a history of infertility for more than one year, observing an improvement in seminal parameters accompanied by higher catalase activity levels after 24 weeks of training which remained significant during the 30 days following the completion of this research. The number of pregnancies, as well as that of live new-borns, grew as well [76]. 

Similarly, Maleki and Tartibian studied the effect of (doing) resistance exercise over male fertility in individuals with a history of infertility extending to more than one year [77]. In accordance with previous studies [75,76], they observed improved seminal parameters that could be significantly correlated with an increase in CAT activity. However, the CAT levels identified in this study reached baseline levels after the post-exercise recovery period instead of remaining stable [77]. The same as in previous similar studies [76], an increase in the rate of pregnancy and live new-borns took place in the study group. 

Not only physical activity can benefit male fertility, adequate rest is important too. Melatonin (*N*-acetyl-5-methoxytryptamine)—a hormone mainly synthesised in the pineal gland of all mammals—regulates the circadian rhythm [78]. When it comes to mammals, the highest levels of melatonin in the blood appear during sleep; hence why a wide range of sleep and mood disorders, jet lag and shift worker conditions are related to an imbalanced melatonin secretion and/or production [79]. Melatonin can be categorised as a very powerful endogenous antioxidant [78]; interestingly, its proved antioxidant properties improve the male reproductive dysfunctions associated both with pathological conditions and with the exposure to toxicants [79]. Rocha and co-workers analysed the different action mechanisms of melatonin in relation to male fertility and highlighted its ability to act directly as a scavenger of different types of free radicals, among them H_2_O_2_, or indirectly through the activation of endogenous antioxidant enzymes such as CAT. Melatonin could thus be an excellent candidate to prevent and/or treat many of the male reproductive dysfunctions linked to various pathologies [79].

Finally, related to psychological stress is relevant to highlight that few studies have explored the relationship between this factor and semen quality. Analysing psychological stress and seminal quality is a matter of great difficulty, since the results reported at the time of writing this work have been found from clinical trials impossible to compare due to the heterogeneity of the samples and the aim of the research conducted. Thus, in some of the studies the stress and anxiety level are analysed in IVF patients (infertile patients) [80,81], whilst other studies were conducted with healthy volunteers [82]. In these investigations, important factor like the age of the patients or the nature of psychological aspects analysed were not considered. Consequently, the literature focused on this topic is still controversial and difficult to compare.

### 3.4. Main Findings Obtained after Reviewing the Clinical Trials Which Study the Relationship between Catalase Levels and Male Fertility 

The review of all the documents selected—and particularly of the clinical trials—made it possible to identify the relationship between CAT activity and male fertility. However, it should be noted that most of the studies focused on this relationship use animal models, which led us to discard them after applying the exclusion criteria; this left with a small number of articles which analyse this relationship in humans. Eight of the 18 articles finally selected—based on the examination of catalase activity in relation to male fertility in humans—were classified as clinical trials. Table 5 below provides a summary of the key points dealt with in those studies, so that their understanding and discussion can become easier.

Although most studies about humans have established a significant relationship between increased CAT activity and seminal quality improvements, note that such studies are based on relatively small sample loads, so that they could have the same variability as a seminogram; to which must be added that only one of the studies took this variability into account, establishing the prudential measure of conducting 3 seminograms—instead of only one—prior to the beginning of the study, thus seeking to minimise the effect of possible spontaneous variations [60].

Furthermore, although the ages of the individuals who took part in the studies were similar, the physiological conditions under examination changed from one study to another. While some considered infertile subjects [60,61,76,77], other studies started from groups of fertile men [62,74] or even from subjects with conditions previously characterised as OS promoters, including alcohol intake or prostatitis [66,70]. Another important aspect worth highlighting about these studies is the fact that the investigations based their seminal diagnoses on different versions of the WHO laboratory manual for the seminal sample processing. In other words, diagnosis heterogeneity is even more frequent than initially expected (subjects characterised as OAT/infertile could be characterised as fertile according to the other criteria when comparing studies due to the updates made in the reference values for seminal diagnosis throughout the years). 

Regarding to the methodologies used to determine catalase activity in biological samples, it turned out that most of the clinical trials performed had followed the same methodology for their study of seminal plasma: the measurement of H_2_O_2_ degradation by spectrophotometry. However, those studies which used blood—instead of seminal plasma—as a biological fluid to measure CAT activity opted for different methodologies. Seminal quality was not studied in these cases and, therefore, the relationship between CAT levels and male fertility remained unknown [66,70]. 

Finally, it should be stressed that, although a strong relationship became visible between variations in CAT activity and seminal quality, this antioxidant only served as a control measure in all these studies, and its correlation with seminal quality variations was actually not studied in every clinical trial.

## 4. Conclusions

The identification of adequate molecular markers for the diagnosis of a semen sample, in conjunction with the design of appropriate therapeutic strategies aimed at combatting defects and deficiencies, becomes decisive to overcome male factor problems. Among them, oxidative stress markers stand out as relevant factors in male fertility, since they play a significant role in sperm physiology. A fine balance of those markers could ultimately determine reproductive success. More specifically, and due to its proven relationship with fertility and normal sperm genesis, the study of catalase activity in seminal samples constitutes a promising field for progress when studying male infertility. 

**Figure 2:**
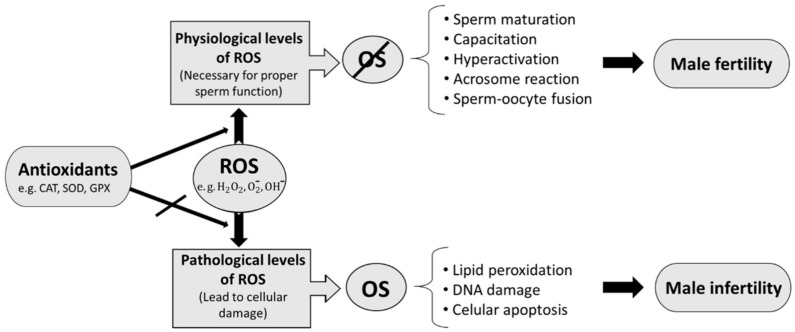
Summary of the interactions between enzymatic antioxidants (catalase as the main one in this work), oxidative stress and male infertility.

Catalase has been shown to play an important role in male fertility. Its antioxidant activity avoids the increase of oxidative stress which can lead to damage in sperm level, and accordingly, to decreased fertility (Figure 2). However, even though consensus exists among most authors on the relationship of this scavenger with male fertility, it has only been studied to a very limited extent in this field.

Most research is carried out on animal models and can therefore not be extrapolated to other species due to the lack of knowledge of the physiological differences that contribute to interspecies variation between man and animals; besides, human studies are actually scarce and lack homogeneity in the methodology utilised. Moreover, the different studies use insufficient and highly disparate sample loads. Nevertheless, despite the shortcomings in the methodology followed in the available studies, they largely agree on highlighting the existence of a significant correlation between the increase of this antioxidant activity and better seminal quality, even in some cases that show significant increases in pregnancy rates. A relationship between scarce catalase activity and a deficient seminal quality became visible too. In any case, despite having verified this relationship, catalase activity is studied together with many other antioxidants in all the studies analysed, without highlighting its specific action and relationship with male fertility among other molecules.

To our knowledge, there are no studies that are correlated with the activity of catalase, neither on the specific parameters of the semen, nor on the level of correlations. At the time of carrying out this review, it was not possible to find previous studies focused on the antioxidant activity of catalase as a direct male fertility biomarker—despite its apparent correlation. For this reason, it seems necessary/advisable to carry out a study meant to analyse the relationship between the variation in catalase activity and male fertility by means of a clear and appropriate methodology for this purpose. Comparing different methodologies in order to finally determine the most appropriate one for this purpose looks like a sensible strategy. Consequently, more research must be addressed in the near future.

If the afore-mentioned correlation ends up being unquestionably verified, it would prove highly useful to introduce the determination of catalase in seminal samples of patients that form part of an assisted reproduction programme. That would permit to observe whether this marker can be considered indicative of good fertilising capacity, as well as of the success achieved in the assisted reproduction technique. Should this be the case, it would mean that a new simple and cost-efficient diagnostic tool for male fertility—complementary to the routine tests included in the study of clinical seminal samples of men with idiopathic infertility—is available.

## Figures and Tables

**Table 1:** Number of publications in each database according to the different keywords utilised, after applying the inclusion criteria.

Keyword	PubMed	Web of Science	Scopus
Catalase	1620	2708	2880
Male fertility	897	1447	1393
Oxidative stress	32,933	56,578	37,182
Catalase & male fertility	1	6	17
Catalase & oxidative stress	862	1,523	1,668
Male fertility & oxidative stress	54	138	136

**Table 2:** Number of publications included in the database for this work (articles identified after the Web of Science search) before and after applying the inclusion criteria and framed within the reproductive biology research area.

Keyword	Total Number of Publications	After IC *	IC and Reproductive Biology (%) **
Catalase & male fertility	82	6	5 (6.1%)
Catalase & oxidative stress	39,712	1515	86 (0.2%)
Male fertility & oxidative stress	667	136	106 (15.9%)

* IC: Inclusion criteria. ** %: calculated over the total number of publications compiled from each keyword combination.

**Table 3:** Number of publications that meet the exclusion criteria because of its main topic.

Publication Main Topic	Number of Publications
Publications including non-human study models	36
Publications about female fertility or embryo development	23
Publications unrelated to fertility	29

**Table 4:** Summary of the authors, countries and institutions significantly contributing to this field of knowledge according to the documents selected for the database created for this study. %: calculated over the total number of publications compiled from each combination of keywords (“MF & OS” = 91; “CAT & MF” = 3; “CAT & OS” = 15). Univ. = University.

Rank	Author			Country			Institution		
	CAT & MF	CAT & OS	MF & OS	CAT & MF	CAT & OS	MF & OS	CAT & MF	CAT & OS	MF & OS
1st	Czerniecki, J. (25%)	Maleki, BH. (17%)	Agarwal, A. (13%)	Japan (33%)	Iran (33%)	USA (26%)	Instituto Valenciano Infertilidad (25%)	Allameh Tabatabai Univ. (17%)	Cleveland Clinic Foundation. (14%)
2nd	Fujii, J. (25%)	Tartibian, B. (17%)	Alves, MG. (7%)	Poland (33%)	Germany (17%)	Italy (12%)	Polish Academy of Sciences (25%)	Justus Liebig Univ. (17%)	Univ. Beira Interior (6%)
3rd	Garrido, N. (25%)	Agarwal, A. (11%)	Oliveira, PF. (7%)	Spain (33%)	USA (17%)	India (11%)	Medical Univ. of Bialystok (25%)	ACECR (11%)	Univ. Do Porto (5%)
4th	Ishii, T. (25%)	Abdollahi, M. (6%)	Rato, L. (4%)		Brazil (11%)	Portugal (11%)		Cleveland clinic foundation (11%)	Mcgill Univ. (4%)
5th	Iuchi, Y. (25%)	Aitken, RJ. (6%)	Aitken, RJ. (3%)		China (11%)	Australia (7%)		Tehran Univ. of Medical Sciences (11%)	Sapienza Univ. Rome (4%)

**Table 5:** Summary of the clinical trials studied.

Ref.	Biological Matrix	CAT Measurement Method	Results	Clinical Implication	Subject of Study
[60]	Seminal plasma	Measurement of H_2_O_2_ degradation by spectrophotometry [83]	Significant CAT increase. Correlation with significant improvement in seminal parameters.	Beneficial effect of PTX on antioxidant capacity of seminal plasma related to improved seminal parameters	Infertile men oligoastenozoospermic (OAT) (25-to-40 years old)
[61]	Seminal plasma	Measurement of H_2_O_2_ degradation by spectrophotometry [84]	Significant CAT increase. No correlation exists between CAT and an improvement in seminal parameters.	Supplementation with Q10 attenuates OS in seminal plasma and allows improvement of antioxidant enzyme activity	Infertile men OAT (25-to-40 years old)
[62]	Seminal plasma	Measurement of H_2_O_2_ degradation by spectrophotometry [84]	Significant CAT increase. Correlation with a significant improvement in seminal parameters.	Honey supplementation reduces the increase in OS during high-intensity exercise and increases CAT levels.	Fertile and unmarried men (18-to-28 years old)
[66]	Blood (erythrocytes)	Measurement of H_2_O_2_ degradation by spectrophotometry [85]	Significant CAT increase. Correlation with seminal parameters not studied.	Bacterial prostatitis causes a decrease of CAT and an increase in ROS resulting in OS damage.	Men with chronic bacterial prostatitis for 1-to-12 years (21-to-30 years old)
[70]	Blood (supernatant and plasma)	Commercial kit	Significant CAT increase. Correlation with seminal parameters not studied.	Continued excessive intake of alcohol results in lower CAT activity and increases OS eventually leading to problems in male fertility.	Alcoholic men (20-to-40 years old)
[75]	Seminal plasma	Measurement of H_2_O_2_ degradation by spectrophotometry [84]	Significant CAT increase. Correlation with a significant improvement in seminal parameters.	Exercise causes improved antioxidant levels, a decrease in OS and better seminal quality, moderate continued exercise representing the best modality.	Unfertile men with sedentary lifestyles (25-to-40 years old)
[76]	Seminal plasma	Measurement of H_2_O_2_ degradation by spectrophotometry [84]	Significant CAT increase. Correlation with a significant improvement in seminal parameters.	Aerobic and anaerobic exercise results in improved CAT levels, decreased OS and enhanced semen quality.	Infertile married men with sedentary lifestyles (25-to-40 years old)
[77]	Seminal plasma	Measurement of H_2_O_2_ degradation by spectrophotometry [84]	Significant CAT increase. Correlation with a significant improvement in seminal parameters.	Conducting resistance exercise causes an improvement in CAT levels, a decrease in OS, and an improvement in semen quality.	Infertile married men with sedentary lifestyles (25-40 years old)

## Supplementary Materials

The following are available online at https://www.mdpi.com/2076-3921/9/1/78/s1, Table S1: Articles included in the database after application of the inclusion and exclusion criteria. Table S2: Articles included in the final database that study the relationship between catalase activity variation and male fertility.
