# Supplementary material for: Catalase as a Molecular Target for Male Infertility Diagnosis and Monitoring: An Overview
Source: Antioxidants (Basel). 2020 Jan 16;9(1):78. doi: 10.3390/antiox9010078 (PMC7022443; doi:10.3390/antiox9010078)
Supplement: Supplementary file 1 [file antioxidants-09-00078-s001.pdf]

Catalase as molecular target for male infertility diagnosis and monitoring: an overview.

Nuria Rubio, Natalia Huerta, María José Gómez, Rosa María Martínez-Espinosa

Antioxidants

Supplementary Table S1. Articles included in the database after application of the inclusion and exclusion criteria.

Title Primary	Authors, Primary	Journal	Pub Year	ISSN/ISBN	DOI
A Randomized Controlled Trial of Lycopene Treatment on Soluble Receptor for Advanced Glycation End Products in Seminal and Blood Plasma of Normospermic Men	Oborna, Ivana; Malickova, Karin; Fingerova, Helena; Brezinova, Jana; Horka, Petra; Novotny, Jiri; Bryndova, Hana; Filipcikova, Radka; Svobodova, Magda	American Journal of Reproductive Immunology	2011	1046-7408	10.1111/j.1600-0897.2011.00984.x
A search for molecular mechanisms underlying male idiopathic infertility	Bracke, An; Peeters, Kris; Punjabi, Usha; Hoogewijs, David; Dewilde, Sylvia	Reproductive Biomedicine Online	2018	1472-6483	10.1016/j.rbmo.2017.12.005
Alcohol abuse-duration dependent decrease in plasma testosterone and antioxidants in males.	Maneesh, M.; Dutta, Sanjiba; Chakrabarti, Amit; Vasudevan, D. M.	Indian journal of physiology and pharmacology	2006	0019-5499	
An updated systematic review on the possible effect of nonylphenol on male fertility	Noorimotlagh, Zahra; Haghighi, Neemat Jaafarzadeh; Ahmadimoghadam, Mehdi; Rahim, Fakher	Environmental Science and Pollution Research	2017	0944-1344	10.1007/s11356-016-7960-y
Antioxidant strategies in the epididymis	Vernet, P.; Aitken, R. J.; Drevet, J. R.	Molecular and cellular endocrinology	2004	0303-7207	10.1016/j.mce.2003.10.069
Antioxidants and sperm DNA damage: a clinical perspective	Zini, Armand; Gabriel, Maria San; Baazeem, Abdulaziz	Journal of assisted reproduction and genetics	2009	1058-0468	10.1007/s10815-009-9343-5
Association of state and trait anxiety to semen quality of in vitro fertilization patients: a controlled study	Vellani, Elisa; Colasante, Alessandro; Mamazza, Luciana; Minasi, Maria Giulia; Greco, Ermanno; Bevilacqua, Arturo	Fertility and sterility	2013	0015-0282	10.1016/j.fertnstert.2013.01.098
Avoiding Toxins Including Spermatotoxic Medications	Stearns, Gillian; Turek, Paul J.	Seminars in reproductive medicine	2013	1526-8004	10.1055/s-0033-1345276
Biomarkers for Male Reproductive health hazards: Are they available?	Ong, C. N.; Shen, H. M.; Chia, S. E.	Toxicology letters	2002	0378-4274	10.1016/S0378-4274(02)00159-5
Cell Phones and Male Infertility: A Review of Recent Innovations in Technology and Consequences	Agarwal, Ashok; Singh, Aspinder; Hamada, Alaa; Kesari, Kavindra	International Braz J Urol	2011	1677-5538	10.1590/S1677-55382011000400002
Cell phones: modern man's nemesis?	Makker, Kartikeya; Varghese, Alex; Desai, Nisarg R.; Mouradi, Rand; Agarwal, Ashok	Reproductive Biomedicine Online	2009	1472-6483	10.1016/S1472-6483(10)60437-3
Chromosomal aberrations, Yq microdeletion, and sperm DNA fragmentation in infertile men opting for assisted reproduction	Shamsi, Monis B.; Kumar, Rajeev; Malhotra, Neena; Singh, Nita; Mittal, Suneeta; Upadhyay, Ashish D.; Dada, Rima	Molecular reproduction and development	2012	1040-452X	10.1002/mrd.22072
Combined aerobic and resistance exercise training for improving reproductive function in infertile men: a randomized controlled trial	Maleki, Behzad Hajizadeh; Tartibian, Bakhtyar	Applied Physiology Nutrition and Metabolism	2017	1715-5312	10.1139/apnm-2017-0249
Contemporary evidence on the physiological role of reactive oxygen species in human sperm function	Du Plessis, Stefan S.; Agarwal, Ashok; Halabi, Jacques; Tvrda, Eva	Journal of assisted reproduction and genetics	2015	1058-0468	10.1007/s10815-014-0425-7
Contribution of sperm molecular features to embryo quality and assisted reproduction success	Garrido, Nicolas; Remohi, Jose; Antonio Martinez-Conejero, Jose; Garcia-Herrero, Sandra; Pellicer, Antonio; Meseguer, Marcos	Reproductive Biomedicine Online	2008	1472-6483	10.1016/S1472-6483(10)60415-4

Cooperative function of antioxidant and redox systems against oxidative stress in male reproductive tissues	Fujii, J.; Iuchi, Y.; Matsuki, S.; Ishii, T.	Asian Journal of Andrology	2003	1008-682X	
Cytokines in the male reproductive tract and their role in infertility disorders	Fraczek, Monika; Kurpisz, Maciej	Journal of reproductive immunology	2015	0165-0378	10.1016/j.jri.2015.02.001
Detecting and Minimizing Sperm DNA Damage	Zhang, Yachao; Trussell, J. C.; Chohan, Kazim R.	Seminars in reproductive medicine	2013	1526-8004	10.1055/s-0033-1345274
Detection of oxidative DNA damage in human sperm and its association with sperm function and male infertility	Shen, H. M.; Ong, C. N.	Free Radical Biology and Medicine	2000	0891-5849	10.1016/S0891-5849(99)00234-8
Diabetes-induced hyperglycemia impairs male reproductive function: a systematic review	Maresch, Constanze C.; Stute, Dina C.; Alves, Marco G.; Oliveira, Pedro F.; de Kretser, David M.; Linn, Thomas	Human reproduction update	2018	1355-4786	10.1093/humupd/dmx033
Diagnosis and impact of sperm DNA alterations in assisted reproduction	Simon, Luke; Emery, Benjamin R.; Carrell, Douglas T.	Best Practice & Research Clinical Obstetrics & Gynaecology	2017	1521-6934	10.1016/j.bpobgyn.2017.07.003
Dietary patterns, foods and nutrients in male fertility parameters and fecundability: a systematic review of observational studies	Salas-Huetos, Albert; Bullo, Monica; Salas-Salvado, Jordi	Human reproduction update	2017	1355-4786	10.1093/humupd/dmx006
Effect of Coenzyme Q10 supplementation on antioxidant enzymes activity and oxidative stress of seminal plasma: a double-blind randomised clinical trial	Nadjarzadeh, A.; Shidfar, F.; Amirjannati, N.; Vafa, M. R.; Motevalian, S. A.; Gohari, M. R.; Kakhki, S. A. Nazeri; Akhondi, M. M.; Sadeghi, M. R.	Andrologia	2014	0303-4569	10.1111/and.12062
Effect of pentoxifylline on semen parameters, reproductive hormones, and seminal plasma antioxidant capacity in men with idiopathic infertility: a randomized double-blind placebo-controlled study	Safarinejad, Mohammad Reza	International urology and nephrology	2011	0301-1623	10.1007/s11255-010-9826-4
Effects of N-acetylcysteine on Semen Parameters and Oxidative/Antioxidant Status	Ciftci, Halil; Verit, Ayhan; Savas, Murat; Yeni, Ercan; Erel, Ozcan	Urology	2009	0090-4295	10.1016/j.urology.2009.02.034
Effects of oral antioxidant treatment upon the dynamics of human sperm DNA fragmentation and subpopulations of sperm with highly degraded DNA	Abad, C.; Amengual, M. J.; Gosalvez, J.; Coward, K.; Hannaoui, N.; Benet, J.; Garcia-Peiro, A.; Prats, J.	Andrologia	2013	0303-4569	10.1111/and.12003
Environmental and occupational pesticide exposure and human sperm parameters: A systematic review	Martenies, Sheena E.; Perry, Melissa J.	Toxicology	2013	0300-483X	10.1016/j.tox.2013.02.005
Falling sperm counts twenty years on: where are we now?	Aitken, R. John	Asian Journal of Andrology	2013	1008-682X	10.1038/aja.2012.167
Fertility and Sperm Quality in the Aging Male	Almeida, Susana; Rato, Luis; Sousa, Mario; Alves, Marco G.; Oliveira, Pedro F.	Current pharmaceutical design	2017	1381-6128	10.2174/1381612823666170503150313
Fluoride Toxicity in the Male Reproductive System	Long, Hu; Jin, Ying; Lin, Mu; Sun, Yu; Zhang, Liang; Clinch, Carole	Fluoride	2009	0015-4725	
Free Radical Theory of Aging: Implications in Male Infertility	Desai, Nisarg; Sabanegh, Edmund, Jr.; Kim, Taesoo; Agarwal, Ashok	Urology	2010	0090-4295	10.1016/j.urology.2009.05.025
Genetic and molecular diagnostics of male infertility in the clinical practice	Pizzol, Damiano; Ferlin, Alberto; Garolla, Andrea; Lenzi, Andrea; Bertoldo, Alessandro; Foresta, Carlo	Frontiers in Bioscience-Landmark	2014	1093-9946	10.2741/4208
Heat Shock Protein A2 (HSPA2): Regulatory Roles in Germ Cell Development and Sperm Function	Nixon, Brett; Bromfield, Elizabeth G.; Cui, Jinwei; De Iuliis, Geoffry N.	Role of Heat Shock Proteins in Reproductive System Development and Function	2017	0301-5556; 978-3-319-51409-3; 978-3-319-51408-6	10.1007/978-3-319-51409-3_4
High-energy diets: a threat for male fertility?	Rato, L.; Alves, M. G.; Cavaco, J. E.; Oliveira, P. F.	Obesity Reviews	2014	1467-7881	10.1111/obr.12226

How to overcome male infertility after 40: Influence of paternal age on fertility	Belloc, Stephanie; Hazout, Andre; Zini, Armand; Merviel, Philippe; Cabry, Rosalie; Chahine, Hikmat; Copin, Henri; Benkhalifa, Moncef	Maturitas	2014	0378-5122	10.1016/j.maturitas.2014.02.011
Impact of Fungicides on Male Reproductive Health: A Review	Sharma, Aksha; Sharma, Preeti; Sharma, Priyanka; Jasuja, Nakuleshwar D.; Joshi, Suresh C.	Research Journal of Pharmaceutical Biological and Chemical Sciences	2015	0975-8585	
Impact of Oxidative Stress on Male Fertility - a Review	Tvrda, Eva; Knazicka, Zuzana; Bardos, Laszlo; Massanyi, Peter; Lukac, Norbert	Acta Veterinaria Hungarica	2011	0236-6290	10.1556/AVet.2011.034
Increased oxidative stress and oxidative damage associated with chronic bacterial prostatitis	Zhou, J. F.; Xiao, W. Q.; Zheng, Y. C.; Dong, J.; Zhang, S. M.	Asian Journal of Andrology	2006	1008-682X	10.1111/j.1745-7262.2006.00144.x
Influence of reactive oxygen species on human sperm functions and fertilizing capacity including therapeutical approaches	Chen, Shu-jian; Allam, Jean-Pierre; Duan, Yong-gang; Haidl, Gerhard	Archives of Gynecology and Obstetrics	2013	0932-0067	10.1007/s00404-013-2801-4
Insight into oxidative stress in varicocele-associated male infertility: part 1	Agarwal, Ashok; Hamada, Alaa; Esteves, Sandro C.	Nature Reviews Urology	2012	1759-4812	10.1038/nrurol.2012.197
Iron and copper in male reproduction: a double-edged sword	Tvrda, Eva; Peer, Rohan; Sikka, Suresh C.; Agarwal, Ashok	Journal of assisted reproduction and genetics	2015	1058-0468	10.1007/s10815-014-0344-7
Leukocytes and oxidative stress: dilemma for sperm function and male fertility	Henkel, Ralf R.	Asian Journal of Andrology	2011	1008-682X	10.1038/aja.2010.76
Lifestyle and fertility: the influence of stress and quality of life on male fertility	Ilacqua, Alessandro; Izzo, Giulia; Emerenziani, Gian Pietro; Baldari, Carlo; Aversa, Antonio	Reproductive Biology and Endocrinology	2018	1477-7827	10.1186/s12958-018-0436-9
Loss of livestock breeding efficiency due to uncompensable sperm nuclear defects	Evenson, D. P.	Reproduction Fertility and Development	1999	1031-3613	10.1071/RD98023
Low-dose radiation-induced risk in spermatogenesis	Fukunaga, Hisanori; Butterworth, Karl T.; Yokoya, Akinari; Ogawa, Takehiko; Prise, Kevin M.	International journal of radiation biology	2017	0955-3002	10.1080/09553002.2017.1355579
May antioxidant therapy improve sperm parameters of men with persistent oligospermia after retrograde embolization for varicocele?	Galatioto, Giuseppe Paradiso; Gravina, Giovanni Luca; Angelozzi, Giovanni; Sacchetti, Antonia; Innominato, Pier Ferdinando; Pace, Gianna; Ranieri, Guido; Vicentini, Carlo	World journal of urology	2008	0724-4983	10.1007/s00345-007-0218-z
Mechanism, measurement, and prevention of oxidative stress in male reproductive physiology	Agarwal, Ashok; Prabakaran, Sushil A.	Indian journal of experimental biology	2005	0019-5189	
Mechanisms of male infertility: Role of antioxidants	Sheweita, S. A.; Tilmisany, A. M.; Al-Sawaf, H.	Current Drug Metabolism	2005	1389-2002	10.2174/138920005774330594
Mechanisms of the harmful effects of bacterial semen infection on ejaculated human spermatozoa: potential inflammatory markers in semen	Fraczek, Monika; Kurpisz, Maciej	Folia Histochemica Et Cytobiologica	2015	0239-8508	10.5603/FHC.a2015.0019
Melatonin and Male Reproductive Health: Relevance of Darkness and Antioxidant Properties	Rocha, C. S.; Rato, L.; Martins, A. D.; Alves, M. G.; Oliveira, P. F.	Current Molecular Medicine	2015	1566-5240	10.2174/1566524015666150505155530
Melatonin, Advanced Oxidation Protein Products and Total Antioxidant Capacity as Seminal Parameters of Prooxidant-Antioxidant Balance and their Connection with Expression of Metalloproteinases in Context of Male Fertility	Kratz, E. M.; Piwowar, A.	Journal of Physiology and Pharmacology	2017	0867-5910	
Microbiological investigation in male infertility: a practical overview	La Vignera, Sandro; Condorelli, Rosita A.; Vicari, Enzo; Salmeri, Mario; Morgia, Giuseppe; Favilla, Vincenzo; Cimino, Sebastiano; Calogero, Aldo E.	Journal of medical microbiology	2014	0022-2615	10.1099/jmm.0.062968-0

Mitochondria functionality and sperm quality	Amaral, Alexandra; Lourenco, Barbara; Marques, Monica; Ramalho-Santos, Joao	Reproduction	2013	1470-1626	10.1530/REP-13-0178
Molecular mechanisms beyond glucose transport in diabetes-related male infertility	Alves, M. G.; Martins, A. D.; Rato, L.; Moreira, P. I.; Socorro, S.; Oliveira, P. F.	Biochimica Et Biophysica Acta-Molecular Basis of Disease	2013	0925-4439	10.1016/j.bbadis.2013.01.011
Mucuna pruriens improves male fertility by its action on the hypothalamus-pituitary-gonadal axis	Shukla, Kamla Kant; Mahdi, Abbas Ali; Ahmad, Mohammad Kaleem; Shankhwar, Satya Narain; Rajender, Singh; Jaiswar, Shyam Pyari	Fertility and sterility	2009	0015-0282	10.1016/j.fertnstert.2008.09.045
Novel Concepts in Male Infertility	Esteves, Sandro C.; Agarwal, Ashok	International Braz J Urol	2011	1677-5538	10.1590/S1677-55382011000700002
Nutrient Supplementation: Improving Male Fertility Fourfold	Mora-Esteves, Cesar; Shin, David	Seminars in reproductive medicine	2013	1526-8004	10.1055/s-0033-1345277
Obesity and Male Infertility: Role of Fatty Acids in the Modulation of Sperm Energetic Metabolism	Ferramosca, Alessandra; Di Giacomo, Mariangela; Moscatelli, Natalina; Zara, Vincenzo	European Journal of Lipid Science and Technology	2018	1438-7697	10.1002/ejlt.201700451
Obesity, a serious etiologic factor for male subfertility in modern society	Liu, Yue; Ding, Zhide	Reproduction	2017	1470-1626	10.1530/REP-17-0161
Paternal obesity negatively affects male fertility and assisted reproduction outcomes: a systematic review and meta-analysis	Campbell, Jared M.; Lane, Michelle; Owens, Julie A.; Bakos, Hassan W.	Reproductive Biomedicine Online	2015	1472-6483	10.1016/j.rbmo.2015.07.012
Physiological Intra-Cytoplasmic Sperm Injection (PICSI) outcomes after oral pretreatment and semen incubation with myo-inositol in oligoasthenoteratozoospermic men: results from a prospective, randomized controlled trial	Korosi, T.; Barta, C.; Rokob, K.; Torok, T.	European review for medical and pharmacological sciences	2017	1128-3602	
Potential biological role of poly (ADP-ribose) polymerase (PARP) in male gametes	Agarwal, Ashok; Mahfouz, Reda Z.; Sharma, Rakesh K.; Sarkar, Oli; Mangrola, Devna; Mathur, Premendu P.	Reproductive Biology and Endocrinology	2009	1477-7827	10.1186/1477-7827-7-143
Pro-oxidative and anti-oxidative imbalance in human semen and its relation with male fertility	Garrido, N.; Meseguer, M.; Simon, C.; Pellicer, A.; Remohi, J.	Asian Journal of Andrology	2004	1008-682X	
Radiations and male fertility	Kesari, Kavindra Kumar; Agarwal, Ashok; Henkel, Ralf	Reproductive Biology and Endocrinology	2018	1477-7827	10.1186/s12958-018-0431-1
Reactive oxygen species and their influence on stallion semen fertility - a review	Pagl, R.; Aurich, J.; Aurich, C.	Pferdeheilkunde	2006	0177-7726	10.21836/PEM20060217
Reactive oxygen species in human sperm suspensions: production by leukocytes and the generation of NADPH to protect sperm against their effects	Ford, W. C. L.; Whittington, K.; Williams, A. C.	International journal of andrology	1997	0105-6263	
Recent scenario of obesity and male fertility	Shukla, K. K.; Chambial, S.; Dwivedi, S.; Misra, S.; Sharma, P.	Andrology	2014	2047-2919	10.1111/andr.270
Redox regulation of fertilisation and the spermatogenic process	Fujii, Junichi; Tsunoda, Satoshi	Asian Journal of Andrology	2011	1008-682X	10.1038/aja.2011.10
Reduction of the incidence of sperm DNA fragmentation by oral antioxidant treatment	Greco, E.; Iacobelli, M.; Rienzi, L.; Ubaldi, F.; Ferrero, S.; Tesarik, J.	Journal of andrology	2005	0196-3635	10.2164/jandrol.04146
Resistance exercise modulates male factor infertility through anti-inflammatory and antioxidative mechanisms in infertile men: A RCT	Maleki, Behzad Hajizadeh; Tartibian, Bakhtyar	Life Sciences	2018	0024-3205	10.1016/j.lfs.2018.04.039
Review of local herbal compounds found in the Iranian traditional medicine known to optimise male fertility	Nejatbakhsh, F.; Shirbeigi, L.; Rahimi, R.; Abolhassani, H.	Andrologia	2016	0303-4569	10.1111/and.12675
Role of oxidants in male infertility: rationale, significance, and treatment	Agarwal, A.; Saleh, R. A.	Urologic Clinics of North America	2002	0094-0143	10.1016/S0094-0143(02)00081-2

Role of oxidative stress and antioxidants in male infertility	Sikka, S. C.; Rajasekaran, M.; Hellstrom, W. J. G.	Journal of andrology	1995	0196-3635	
Role of reactive oxygen species in male infertility	Sharma, R. K.; Agarwal, A.	Urology	1996	0090-4295	10.1016/S0090-4295(96)00313-5
Role of Withania somnifera (Ashwagandha) in the management of male infertility	Sengupta, Pallav; Agarwal, Ashok; Pogrebetskaya, Maria; Roychoudhury, Shubhadeep; Durairajanayagam, Damayanthi; Henkel, Ralf	Reproductive Biomedicine Online	2018	1472-6483	10.1016/j.rbmo.2017.11.007
Seminal suPAR Levels as Marker of Abacterial Male Accessory Gland Inflammation in Hypogonadism	Milardi, Domenico; Grande, Giuseppe; Autilio, Chiara; Mancini, Francesca; De Marinis, Laura; Marana, Riccardo; Zuppi, Cecilia; Urbani, Andrea; Pontecorvi, Alfredo; Baroni, Silvia	Protein and Peptide Letters	2018	0929-8665	10.2174/0929866525666180418121421
Sperm chromatin structure and male fertility: biological and clinical aspects	Erenpreiss, J.; Spano, M.; Erenpreisa, J.; Bungum, M.; Giwercman, A.	Asian Journal of Andrology	2006	1008-682X	10.1111/j.1745-7262.2006.00112.x
Sperm Cryopreservation: Effects on Chromatin Structure	Paoli, Donatella; Lombardo, Francesco; Lenzi, Andrea; Gandini, Loredana	Genetic Damage in Human Spermatozoa	2014	0065-2598; 978-1-4614-7783-9; 978-1-4614-7782-2	10.1007/978-1-4614-7783-9_9
Sperm DNA integrity assays: diagnostic and prognostic challenges and implications in management of infertility	Shamsi, Monis Bilal; Imam, Syed Nazar; Dada, Rima	Journal of assisted reproduction and genetics	2011	1058-0468	10.1007/s10815-011-9631-8
Sperm function tests and fertility	Aitken, R. J.	International journal of andrology	2006	0105-6263	10.1111/j.1365-2605.2005.00630.x
Sperm glucose transport and metabolism in diabetic individuals	Dias, Tania R.; Alves, Marco G.; Silva, Branca M.; Oliveira, Pedro F.	Molecular and cellular endocrinology	2014	0303-7207	10.1016/j.mce.2014.08.005
Sperm Proteome: What Is on the Horizon?	Mohanty, Gayatri; Swain, Nirlipta; Samanta, Luna	Reproductive Sciences	2015	1933-7191	10.1177/1933719114558918
Spermatozoal sensitive biomarkers to defective protaminosis and fragmented DNA	Angelopoulou, Roxani; Plastira, Konstantina; Msaouel, Pavlos	Reproductive Biology and Endocrinology	2007	1477-7827	10.1186/1477-7827-5-36
The controversial efficacy of vitamin E for human male infertility	Bolle, P.; Evandri, M. G.; Saso, L.	Contraception	2002	0010-7824	10.1016/S0010-7824(02)00277-9
The Effect of Chronic Prostatitis/Chronic Pelvic Pain Syndrome (CP/CPPS) on Semen Parameters in Human Males: A Systematic Review and Meta-Analysis	Fu, Weihua; Zhou, Zhansong; Liu, Shijian; Li, Qianwei; Yao, Jiwei; Li, Weibing; Yan, Junan	Plos One	2014	1932-6203	10.1371/journal.pone.0094991
The effect of cigarette smoking, alcohol consumption and fruit and vegetable consumption on IVF outcomes: a review and presentation of original data	Firns, Sarah; Cruzat, Vinicius Fernandes; Keane, Kevin Noel; Joesbury, Karen A.; Lee, Andy H.; Newsholme, Philip; Yovich, John L.	Reproductive Biology and Endocrinology	2015	1477-7827	10.1186/s12958-015-0133-x
The Effects of Honey Supplementation on Seminal Plasma Cytokines, Oxidative Stress Biomarkers, and Antioxidants During 8 Weeks of Intensive Cycling Training	Tartibian, Bakhtyar; Maleki, Behzad Hajizadeh	Journal of andrology	2012	0196-3635	10.2164/jandrol.110.012815
The effects of three different exercise modalities on markers of male reproduction in healthy subjects: a randomized controlled trial	Maleki, Behzad Hajizadeh; Tartibian, Bakhtyar; Chehrazi, Mohammad	Reproduction	2017	1470-1626	10.1530/REP-16-0318
The hazardous effects of tobacco smoking on male fertility	Dai, Jing-Bo; Wang, Zhao-Xia; Qiao, Zhong-Dong	Asian Journal of Andrology	2015	1008-682X	10.4103/1008-682X.150847
The impact of obesity on male fertility	Chambers, Thomas J. G.; Anderson, Richard A.	Hormones-International Journal of Endocrinology and Metabolism	2015	1109-3099	

The Impact of Shock Wave Lithotripsy on Male Fertility: A Critical Analysis of Existing Evidence	Philippou, Prodromos; Ralph, David J.; Timoney, Anthony G.	Urology	2012	0090-4295	10.1016/j.urology.2011.12.003
The impact of sperm protamine deficiency and sperm DNA damage on human male fertility: a systematic review and meta-analysis	Ni, K.; Spiess, A. -N; Schuppe, H. -C; Steger, K.	Andrology	2016	2047-2919	10.1111/andr.12216
The non-genomic effects of endocrine-disrupting chemicals on mammalian sperm	Tavares, R. S.; Escada-Rebelo, S.; Correia, M.; Mota, P. C.; Ramalho-Santos, J.	Reproduction	2016	1470-1626	10.1530/REP-15-0355
The role of antioxidant therapy in the treatment of male infertility: an overview	Lombardo, Francesco; Sansone, Andrea; Romanelli, Francesco; Paoli, Donatella; Gandini, Loredana; Lenzi, Andrea	Asian Journal of Andrology	2011	1008-682X	10.1038/aja.2010.183
The Role of Nutraceuticals in Male Fertility	Ko, Edmund Y.; Sabanegh, Edmund S.	Urologic Clinics of North America	2014	0094-0143	10.1016/j.ucl.2013.08.003
The Role of Over-the-Counter Supplements for the Treatment of Male Infertility-Fact or Fiction?	Ko, Edmund Y.; Sabanegh, Edmund S., Jr.	Journal of andrology	2012	0196-3635	10.2164/jandrol.111.013730
The role of oxidative stress and antioxidants in male fertility.	Walczak-Jedrzejowska, Renata; Wolski, Jan Karol; Slowikowska-Hilczer, Jolanta	Central European journal of urology	2013	2080-4806	10.5173/ceju.2013.01.art19
The role of sperm oxidative stress in male infertility and the significance of oral antioxidant therapy	Gharagozloo, Parviz; Aitken, R. John	Human Reproduction	2011	0268-1161	10.1093/humrep/der132
The sperm mitochondrion: Organelle of many functions	Moraes, Christa R.; Meyers, Stuart	Animal Reproduction Science	2018	0378-4320	10.1016/j.anireprosci.2018.03.024
The toxicology of indium tin oxide	Bomhard, Ernst M.	Environmental toxicology and pharmacology	2016	1382-6689	10.1016/j.etap.2016.06.011
TNF-alpha -308 polymorphisms and male infertility risk: A meta-analysis and systematic	Mostafa, Taymour; Taymour, Mai	Journal of Advanced Research	2016	2090-1232	10.1016/j.jare.2015.07.001
Toxicants and human sperm chromatin integrity	Delbes, Geraldine; Hales, Barbara F.; Robaire, Bernard	Molecular human reproduction	2010	1360-9947	10.1093/molehr/gap087
Unravelling the Power of Omics for the Infertile Aging Male	Bastos, Paulo; Freitas, Maria Joao; Gomes, Andre; Vitorino, Rui; Fardilha, Margarida	Current pharmaceutical design	2017	1381-6128	10.2174/1381612822666161018155247
Using the alkaline comet assay in prognostic tests for male infertility and assisted reproductive technology outcomes	Lewis, Sheena E. M.; Agbaje, Ishola M.	Mutagenesis	2008	0267-8357	10.1093/mutage/gem052
Variations in Antioxidant Genes and Male Infertility.	Yu, Bolan; Huang, Zhaofeng	BioMed research international	2015		10.1155/2015/513196
Varicocele and testicular function	Pastuszak, Alexander W.; Wang, Run	Asian Journal of Andrology	2015	1008-682X	10.4103/1008-682X.153539
Varicocele management in the era of in vitro fertilization/intracytoplasmic sperm injection	Pathak, Piyush; Chandrashekar, Aravind; Hakky, Tariq S.; Pastuszak, Alexander W.	Asian Journal of Andrology	2016	1008-682X	10.4103/1008-682X.178482
Varicocele repair: does it still have a role in infertility treatment?	French, Dan B.; Desai, Nisarg R.; Agarwal, Ashok	Current opinion in obstetrics & gynecology	2008	1040-872X	10.1097/GCO.0b013e3282fcc00c
What every gynecologist should know about male infertility: an update	Esteves, Sandro C.; Hamada, Alaa; Kondray, Victor; Pitchika, Aruna; Agarwal, Ashok	Archives of Gynecology and Obstetrics	2012	0932-0067	10.1007/s00404-012-2274-x
Nitric Oxide and Reactive Oxygen Species in the Pathogenesis of Preeclampsia	Matsubara, Keiichi; Higaki, Takashi; Matsubara, Yuko; Nawa, Akihiro	International Journal of Molecular Sciences	2015	1422-0067	10.3390/ijms16034600
Novel Concepts in Male Infertility	Esteves, Sandro C.; Agarwal, Ashok	International Braz J Urol	2011	1677-5538	10.1590/S1677-55382011000700002
Nrf2/ARE regulated antioxidant gene expression in endothelial and smooth muscle cells in oxidative stress: implications for atherosclerosis and preeclampsia.	Mann, Giovanni E.; Niehueser-Saran, Jorg; Watson, Alan; Gao, Ling; Ishii, Tetsuro; de Winter, Patricia; Siow, Richard C.	Sheng li xue bao: [Acta physiologica Sinica]	2007	0371-0874	

Nutrient Supplementation: Improving Male Fertility Fourfold	Mora-Esteves, Cesar; Shin, David	Seminars in reproductive medicine	2013	1526-8004	10.1055/s-0033-1345277
Nutrigenetics and Modulation of Oxidative Stress	Da Costa, Laura A.; Badawi, Alaa; El-Sohemy, Ahmed	Annals of Nutrition and Metabolism	2012	0250-6807	10.1159/000337311
Nutritional and exercise-based interventions in the treatment of amyotrophic lateral sclerosis	Patel, Barkha P.; Hamadeh, Mazen J.	Clinical Nutrition	2009	0261-5614	10.1016/j.clnu.2009.06.002
Obesity and Male Infertility: Role of Fatty Acids in the Modulation of Sperm Energetic Metabolism	Ferramosca, Alessandra; Di Giacomo, Mariangela; Moscatelli, Natalina; Zara, Vincenzo	European Journal of Lipid Science and Technology	2018	1438-7697	10.1002/ejlt.201700451
Obesity, a serious etiologic factor for male subfertility in modern society	Liu, Yue; Ding, Zhide	Reproduction	2017	1470-1626	10.1530/REP-17-0161
Omega-3 LCPUFA supplement: a nutritional strategy to prevent maternal and neonatal oxidative stress	Kajarabille, Naroa; Hurtado, Jose A.; Pena-Quintana, Luis; Pena, Manuela; Ruiz, Josefa; Diaz-Castro, Javier; Rodriguez-Santana, Yessica; Martin-Alvarez, Estefania; Lopez-Frias, Magdalena; Soldado, Olga; Lara-Villoslada, Federico; Ochoa, Julio J.	Maternal and Child Nutrition	2017	1740-8695	10.1111/mcn.12300
Oxidant stress in pre-eclampsia and essential hypertension.	Kumar, C. A.; Das, U. N.	The Journal of the Association of Physicians of India	2002	0004-5772	
Oxidative damage in chemical teratogenesis	Wells, P. G.; Kim, P. M.; Laposa, R. R.; Nicol, C. J.; Parman, T.; Winn, L. M.	Mutation Research-Fundamental and Molecular Mechanisms of Mutagenesis	1997	0027-5107	10.1016/S0027-5107(97)00175-9
Oxidative Stress and Redox Regulation on In Vitro Development of Mammalian Embryos	Takahashi, Masashi	Journal of Reproduction and Development	2012	0916-8818	10.1262/jrd.11-138N
Oxidative Stress and Role of Natural Plant Derived Antioxidants in Animal Reproduction	Zhong Rong-zhen; Zhou Dao-wei	Journal of Integrative Agriculture	2013	2095-3119	10.1016/S2095-3119(13)60412-8
Oxidative stress in diabetic pregnancy: SOD, CAT and GSH-Px activity and lipid peroxidation products.	Djordjevic, A.; Spasic, S.; Jovanovic-Galovic, A.; Djordjevic, R.; Grubor-Lajsic, G.	The journal of maternal-fetal & neonatal medicine: the official journal of the European Association of Perinatal Medicine, the Federation of Asia and Oceania Perinatal Societies, the International Society of Perinatal Obstetricians	2004	1476-7058	
Oxidative Stress in Granulosa-Lutein Cells From In Vitro Fertilization Patients	Avila, Julio; Gonzalez-Fernandez, Rebeca; Rotoli, Deborah; Hernandez, Jairo; Palumbo, Angela	Reproductive Sciences	2016	1933-7191	10.1177/1933719116674077
Oxidative Stress, Antioxidants, and Animal Function	Miller, J. K.; Brzezinskaslebodzinska, E.; Madsen, F. C.	Journal of dairy science	1993	0022-0302	10.3168/jds.S0022-0302(93)77620-1
Paternal obesity negatively affects male fertility and assisted reproduction outcomes: a systematic review and meta-analysis	Campbell, Jared M.; Lane, Michelle; Owens, Julie A.; Bakos, Hassan W.	Reproductive Biomedicine Online	2015	1472-6483	10.1016/j.rbmo.2015.07.012
Pharmacological interventions in aging and age-associated disorders	Kitani, Kenichi	Geriatrics & Gerontology International	2007	1444-1586	10.1111/j.1447-0594.2007.00377.x
Pharmacology of delayed aging and extended lifespan of Caenorhabditis elegans	Collins, James J.; Evason, Kimberley; Kornfeld, Kerry	Experimental gerontology	2006	0531-5565	10.1016/j.exger.2006.06.038
Physiological Intra-Cytoplasmic Sperm Injection (PICSI) outcomes after oral pretreatment and semen incubation	Korosi, T.; Barta, C.; Rokob, K.; Torok, T.	European review for medical and pharmacological sciences	2017	1128-3602	

with myo-inositol in oligoasthenoteratozoospermic men: results from a prospective, randomized controlled trial					
Placental antioxidant enzyme status and lipid peroxidation in pregnant women with type 1 diabetes: The effect of vitamin C and E supplementation	Johnston, Philip C.; McCance, David R.; Holmes, Valerie A.; Young, Ian S.; McGinty, Ann	Journal of diabetes and its complications	2016	1056-8727	10.1016/j.jdiacomp.2015.10.001
Potential biological role of poly (ADP-ribose) polymerase (PARP) in male gametes	Agarwal, Ashok; Mahfouz, Reda Z.; Sharma, Rakesh K.; Sarkar, Oli; Mangrola, Devna; Mathur, Premendu P.	Reproductive Biology and Endocrinology	2009	1477-7827	10.1186/1477-7827-7-143
Probiotic yogurt improves antioxidant status in type 2 diabetic patients	Ejtahed, Hanie S.; Mohtadi-Nia, Javad; Homayouni-Rad, Aziz; Niafar, Mitra; Asghari-Jafarabadi, Mohammad; Mofid, Vahid	Nutrition	2012	0899-9007	10.1016/j.nut.2011.08.013
Pro-oxidative and anti-oxidative imbalance in human semen and its relation with male fertility	Garrido, N.; Meseguer, M.; Simon, C.; Pellicer, A.; Remohi, J.	Asian Journal of Andrology	2004	1008-682X	
Radiations and male fertility	Kesari, Kavindra Kumar; Agarwal, Ashok; Henkel, Ralf	Reproductive Biology and Endocrinology	2018	1477-7827	10.1186/s12958-018-0431-1
Reactive oxygen species and their influence on stallion semen fertility - a review	Pagl, R.; Aurich, J.; Aurich, C.	Pferdeheilkunde	2006	0177-7726	10.21836/PEM20060217
Reactive oxygen species in human sperm suspensions: production by leukocytes and the generation of NADPH to protect sperm against their effects	Ford, W. C. L.; Whittington, K.; Williams, A. C.	International journal of andrology	1997	0105-6263	
Recent scenario of obesity and male fertility	Shukla, K. K.; Chambial, S.; Dwivedi, S.; Misra, S.; Sharma, P.	Andrology	2014	2047-2919	10.1111/andr.270
Red blood cell glutathione peroxidase activity in female nulligravid and pregnant rats	Gallo, Giuseppe; Martino, Guglielmo	Reproductive Biology and Endocrinology	2009	1477-7827	10.1186/1477-7827-7-7
Redox regulation of fertilisation and the spermatogenic process	Fujii, Junichi; Tsunoda, Satoshi	Asian Journal of Andrology	2011	1008-682X	10.1038/aja.2011.10
Reduction of the incidence of sperm DNA fragmentation by oral antioxidant treatment	Greco, E.; Iacobelli, M.; Rienzi, L.; Ubaldi, F.; Ferrero, S.; Tesarik, J.	Journal of andrology	2005	0196-3635	10.2164/jandrol.04146
Resistance exercise modulates male factor infertility through anti-inflammatory and antioxidative mechanisms in infertile men: A RCT	Maleki, Behzad Hajizadeh; Tartibian, Bakhtyar	Life Sciences	2018	0024-3205	10.1016/j.lfs.2018.04.039
Resveratrol protects quail hepatocytes against heat stress: modulation of the Nrf2 transcription factor and heat shock proteins	Sahin, K.; Orhan, C.; Akdemir, F.; Tuzcu, M.; Iben, C.; Sahin, N.	Journal of Animal Physiology and Animal Nutrition	2012	0931-2439	10.1111/j.1439-0396.2010.01123.x
Review of local herbal compounds found in the Iranian traditional medicine known to optimise male fertility	Nejatbakhsh, F.; Shirbeigi, L.; Rahimi, R.; Abolhassani, H.	Andrologia	2016	0303-4569	10.1111/and.12675
Review: Diagnosis and impact of sperm DNA alterations in assisted reproduction	Simon, Luke; Emery, Benjamin R.; Carrell, Douglas T.	Best Practice & Research Clinical Obstetrics & Gynaecology	2017	1521-6934	10.1016/j.bpobgyn.2017.07.003
Review: Maternal and placental antioxidant response to preeclampsia - Impact on vasoactive eicosanoids	Bilodeau, J. -F	Placenta	2014	0143-4004	10.1016/j.placenta.2013.11.013
Role of antioxidants in gestational diabetes mellitus and relation to fetal outcome: a randomized controlled trial	Maged, Ahmed M.; Torky, Haitham; Fouad, Mona A.; GadAllah, Sherine H.; Waked, Nevien M.; Gayed, Ahmed S.; Salem, Ashraf K.	Journal of Maternal-Fetal & Neonatal Medicine	2016	1476-7058	10.3109/14767058.2016.1154526
Role of copper in the process of spermatogenesis.	Ogorek, Mateusz; Gasior, Lukasz; Pierzchala, Olga; Daszkiewicz, Regina; Lenartowicz, Malgorzata	Postepy higieny i medycyny doswiadczalnej (Online)	2017		
Role of oxidants in male infertility: rationale, significance, and treatment	Agarwal, A.; Saleh, R. A.	Urologic Clinics of North America	2002	0094-0143	10.1016/S0094-0143(02)00081-2

Role of oxidative stress and antioxidants in male infertility	Sikka, S. C.; Rajasekaran, M.; Hellstrom, W. J. G.	Journal of andrology	1995	0196-3635	
Role of Oxidative Stress in Male Reproductive Dysfunctions with Reference to Phthalate Compounds	Sedha, Sapna; Kumar, Sunil; Shukla, Shruti	Urology Journal	2015	1735-1308	
Role of reactive oxygen species in male infertility	Sharma, R. K.; Agarwal, A.	Urology	1996	0090-4295	10.1016/S0090-4295(96)00313-5
Role of the antioxidants in the protection against oxidative stress in cattle: Nonenzymatic mechanisms (Part 2).	Kleczkowski, M.; Klucinski, W.; Sikora, J.; Zdanowicz, M.; Dziekan, P.	Polish Journal of Veterinary Sciences	2003	1505-1773	
Role of Withania somnifera (Ashwagandha) in the management of male infertility	Sengupta, Pallav; Agarwal, Ashok; Pogrebetskaya, Maria; Roychoudhury, Shubhadeep; Durairajanayagam, Damayanthi; Henkel, Ralf	Reproductive Biomedicine Online	2018	1472-6483	10.1016/j.rbmo.2017.11.007
Sarcopenia is more than a muscular deficit	Fulle, S.; Belia, S.; Di Tano, G.	Archives Italiennes de Biologie	2005	0003-9829	
Seminal suPAR Levels as Marker of Abacterial Male Accessory Gland Inflammation in Hypogonadism	Milardi, Domenico; Grande, Giuseppe; Autilio, Chiara; Mancini, Francesca; De Marinis, Laura; Marana, Riccardo; Zuppi, Cecilia; Urbani, Andrea; Pontecorvi, Alfredo; Baroni, Silvia	Protein and Peptide Letters	2018	0929-8665	10.2174/0929866525666180418121421
Sperm chromatin structure and male fertility: biological and clinical aspects	Erenpreiss, J.; Spano, M.; Erenpreisa, J.; Bungum, M.; Giwercman, A.	Asian Journal of Andrology	2006	1008-682X	10.1111/j.1745-7262.2006.00112.x
Sperm Cryopreservation: Effects on Chromatin Structure	Paoli, Donatella; Lombardo, Francesco; Lenzi, Andrea; Gandini, Loredana	Genetic Damage in Human Spermatozoa	2014	0065-2598; 978-1-4614-7783-9; 978-1-4614-7782-2	10.1007/978-1-4614-7783-9_9
Sperm DNA integrity assays: diagnostic and prognostic challenges and implications in management of infertility	Shamsi, Monis Bilal; Imam, Syed Nazar; Dada, Rima	Journal of assisted reproduction and genetics	2011	1058-0468	10.1007/s10815-011-9631-8
Sperm function tests and fertility	Aitken, R. J.	International journal of andrology	2006	0105-6263	10.1111/j.1365-2605.2005.00630.x
Sperm glucose transport and metabolism in diabetic individuals	Dias, Tania R.; Alves, Marco G.; Silva, Branca M.; Oliveira, Pedro F.	Molecular and cellular endocrinology	2014	0303-7207	10.1016/j.mce.2014.08.005
Sperm Proteome: What Is on the Horizon?	Mohanty, Gayatri; Swain, Nirlipta; Samanta, Luna	Reproductive Sciences	2015	1933-7191	10.1177/1933719114558918
Spermatozoal sensitive biomarkers to defective protaminosis and fragmented DNA	Angelopoulou, Roxani; Plastira, Konstantina; Msaouel, Pavlos	Reproductive Biology and Endocrinology	2007	1477-7827	10.1186/1477-7827-5-36
Supplementation of ram semen extender to improve seminal quality and fertility rate	Allai, Larbi; Benmoula, Anass; Maia, Marciane da Silva; Nasser, Boubker; El Amiri, Bouchra	Animal Reproduction Science	2018	0378-4320	10.1016/j.anireprosci.2018.03.019
Supplementation of vitamin E, selenium and increased energy allowance mitigates the transition stress and improves postpartum reproductive performance in the crossbred cow	Khatti, Amit; Mehrotra, Sanjeev; Patel, Pankaj Kumar; Singh, Gyanendra; Maurya, Vijai Prakash; Mahla, Ajit Singh; Chaudhari, Ravjibhai Karshanbhai; Das, Gautam Kumar; Singh, Mithilesh; Sarkar, Mihir; Kumar, Harendra; Krishnaswamy, Narayanan	Theriogenology	2017	0093-691X	10.1016/j.theriogenology.2017.08.014
Supplementation with copper edetate in control of Haemonchus contortus of sheep, and its effect on cholinesterase's and superoxide dismutase activities	Grosskopf, Hyolanda M.; Grosskopf, Rhayana K.; Biazus, Angelisa H.; Leal, Marta L. R.; Bottari, Nathieli B.; Alves, Mariana S.; Schetinger, Maria Rosa C.; Morsch, Vera M.; Machado, Gustavo; Baldissera, Matheus D.; Da Silva, Aleksandro S.	Experimental parasitology	2017	0014-4894	10.1016/j.exppara.2016.12.011

The Characteristics of Blood Glucose and WBC Counts in Peripheral Blood of Cases of Hand Foot and Mouth Disease in China: A Systematic Review	Li, Yuyun; Zhu, Runan; Qian, Yuan; Deng, Jie	Plos One	2012	1932-6203	10.1371/journal.pone.0029003
The controversial efficacy of vitamin E for human male infertility	Bolle, P.; Evandri, M. G.; Saso, L.	Contraception	2002	0010-7824	10.1016/S0010-7824(02)00277-9
The Effect of Chronic Prostatitis/Chronic Pelvic Pain Syndrome (CP/CPPS) on Semen Parameters in Human Males: A Systematic Review and Meta-Analysis	Fu, Weihua; Zhou, Zhansong; Liu, Shijian; Li, Qianwei; Yao, Jiwei; Li, Weibing; Yan, Junan	Plos One	2014	1932-6203	10.1371/journal.pone.0094991
The effect of cigarette smoking, alcohol consumption and fruit and vegetable consumption on IVF outcomes: a review and presentation of original data	Firns, Sarah; Cruzat, Vinicius Fernandes; Keane, Kevin Noel; Joesbury, Karen A.; Lee, Andy H.; Newsholme, Philip; Yovich, John L.	Reproductive Biology and Endocrinology	2015	1477-7827	10.1186/s12958-015-0133-x
The effect of environmental contaminants on testicular function	Mathur, Premendu Prakash; D'Cruz, Shereen Cynthia	Asian Journal of Andrology	2011	1008-682X	10.1038/aja.2011.40
The Effects of Honey Supplementation on Seminal Plasma Cytokines, Oxidative Stress Biomarkers, and Antioxidants During 8 Weeks of Intensive Cycling Training	Tartibian, Bakhtyar; Maleki, Behzad Hajizadeh	Journal of andrology	2012	0196-3635	10.2164/jandrol.110.012815
The effects of hormone replacement therapy combined with vitamins C and E on antioxidants levels and lipid profiles in postmenopausal women with type 2 diabetes	Naziroglu, M.; Simsek, M.; Simsek, H.; Aydilek, N.; Ozcan, Z.; Atilgan, R.	Clinica Chimica Acta	2004	0009-8981	10.1016/j.cccn.2004.01.031
The effects of three different exercise modalities on markers of male reproduction in healthy subjects: a randomized controlled trial	Maleki, Behzad Hajizadeh; Tartibian, Bakhtyar; Chehrazi, Mohammad	Reproduction	2017	1470-1626	10.1530/REP-16-0318
The etiology of oxidative stress in the various species of animals, a review	Puppel, Kamila; Kapusta, Aleksandra; Kuczynska, Beata	Journal of the science of food and agriculture	2015	0022-5142	10.1002/jsfa.7015
The hazardous effects of tobacco smoking on male fertility	Dai, Jing-Bo; Wang, Zhao-Xia; Qiao, Zhong-Dong	Asian Journal of Andrology	2015	1008-682X	10.4103/1008-682X.150847
The impact of obesity on male fertility	Chambers, Thomas J. G.; Anderson, Richard A.	Hormones-International Journal of Endocrinology and Metabolism	2015	1109-3099	
The Impact of Shock Wave Lithotripsy on Male Fertility: A Critical Analysis of Existing Evidence	Philippou, Prodromos; Ralph, David J.; Timoney, Anthony G.	Urology	2012	0090-4295	10.1016/j.urology.2011.12.003
The impact of sperm protamine deficiency and sperm DNA damage on human male fertility: a systematic review and meta-analysis	Ni, K.; Spiess, A. -N; Schuppe, H. -C; Steger, K.	Andrology	2016	2047-2919	10.1111/andr.12216
The influence of age and gender on antioxidant enzyme activities in humans and laboratory animals	Giergiel, Marta; Lopucki, Maciej; Stachowicz, Norbert; Kankofer, Marta	Aging Clinical and Experimental Research	2012	1594-0667	10.3275/8587
The non-genomic effects of endocrine-disrupting chemicals on mammalian sperm	Tavares, R. S.; Escada-Rebelo, S.; Correia, M.; Mota, P. C.; Ramalho-Santos, J.	Reproduction	2016	1470-1626	10.1530/REP-15-0355
The Protective Effects of Different Sources of Maternal Selenium on Oxidative Stressed Chick Embryo Liver	Xiao, Xue; Yuan, Dong; Wang, Yong-Xia; Zhan, Xiu-An	Biological trace element research	2016	0163-4984	10.1007/s12011-015-0541-y
The role of antioxidant therapy in the treatment of male infertility: an overview	Lombardo, Francesco; Sansone, Andrea; Romanelli, Francesco; Paoli, Donatella; Gandini, Loredana; Lenzi, Andrea	Asian Journal of Andrology	2011	1008-682X	10.1038/aja.2010.183
The Role of Nutraceuticals in Male Fertility	Ko, Edmund Y.; Sabanegh, Edmund S.	Urologic Clinics of North America	2014	0094-0143	10.1016/j.ucl.2013.08.003
The Role of Over-the-Counter Supplements for the Treatment of Male Infertility-Fact or Fiction?	Ko, Edmund Y.; Sabanegh, Edmund S., Jr.	Journal of andrology	2012	0196-3635	10.2164/jandrol.111.013730

The role of oxidative stress and antioxidants in male fertility.	Walczak-Jedrzejowska, Renata; Wolski, Jan Karol; Slowikowska-Hilczer, Jolanta	Central European journal of urology	2013	2080-4806	10.5173/ceju.2013.01.art19
The role of sperm oxidative stress in male infertility and the significance of oral antioxidant therapy	Gharagozloo, Parviz; Aitken, R. John	Human Reproduction	2011	0268-1161	10.1093/humrep/der132
The roles of cellular reactive oxygen species, oxidative stress and antioxidants in pregnancy outcomes	Al-Gubory, Kais H.; Fowler, Paul A.; Garrel, Catherine	International Journal of Biochemistry & Cell Biology	2010	1357-2725	10.1016/j.biocel.2010.06.001
The sperm mitochondrion: Organelle of many functions	Moraes, Christa R.; Meyers, Stuart	Animal Reproduction Science	2018	0378-4320	10.1016/j.anireprosci.2018.03.024
The toxicology of indium tin oxide	Bomhard, Ernst M.	Environmental toxicology and pharmacology	2016	1382-6689	10.1016/j.etap.2016.06.011
TNF-alpha -308 polymorphisms and male infertility risk: A meta-analysis and systematic	Mostafa, Taymour; Taymour, Mai	Journal of Advanced Research	2016	2090-1232	10.1016/j.jare.2015.07.001
Toxicants and human sperm chromatin integrity	Delbes, Geraldine; Hales, Barbara F.; Robaire, Bernard	Molecular human reproduction	2010	1360-9947	10.1093/molehr/gap087
Unravelling the Power of Omics for the Infertile Aging Male	Bastos, Paulo; Freitas, Maria Joao; Gomes, Andre; Vitorino, Rui; Fardilha, Margarida	Current pharmaceutical design	2017	1381-6128	10.2174/1381612822666161018155247
Using the alkaline comet assay in prognostic tests for male infertility and assisted reproductive technology outcomes	Lewis, Sheena E. M.; Agbaje, Ishola M.	Mutagenesis	2008	0267-8357	10.1093/mutage/gem052
Variations in Antioxidant Genes and Male Infertility.	Yu, Bolan; Huang, Zhaofeng	BioMed research international	2015		10.1155/2015/513196
Varicocele and testicular function	Pastuszak, Alexander W.; Wang, Run	Asian Journal of Andrology	2015	1008-682X	10.4103/1008-682X.153539
Varicocele management in the era of in vitro fertilization/intracytoplasmic sperm injection	Pathak, Piyush; Chandrashekar, Aravind; Hakky, Tariq S.; Pastuszak, Alexander W.	Asian Journal of Andrology	2016	1008-682X	10.4103/1008-682X.178482
Varicocele repair: does it still have a role in infertility treatment?	French, Dan B.; Desai, Nisarg R.; Agarwal, Ashok	Current opinion in obstetrics & gynecology	2008	1040-872X	10.1097/GCO.0b013e3282fcc00c
What every gynecologist should know about male infertility: an update	Esteves, Sandro C.; Hamada, Alaa; Kondray, Victor; Pitchika, Aruna; Agarwal, Ashok	Archives of Gynecology and Obstetrics	2012	0932-0067	10.1007/s00404-012-2274-x

Catalase as molecular target for male infertility diagnosis and monitoring: an overview.

Nuria Rubio, Natalia Huerta, María José Gómez, Rosa María Martínez-Espinosa

Antioxidants

Supplementary Table S2. Articles included in the final database that study the relationship between catalase activity variation and male fertility.

Title Primary	Authors, Primary	Journal	Pub Year	ISSN/ISBN	DOI
Alcohol abuse-duration dependent decrease in plasma testosterone and antioxidants in males.	Maneesh, M.; Dutta, Sanjiba; Chakrabarti, Amit; Vasudevan, D. M.	Indian journal of physiology and pharmacology	2006	0019-5499	
Antioxidants and sperm DNA damage: a clinical perspective	Zini, Armand; Gabriel, Maria San; Baazeem, Abdulaziz	Journal of assisted reproduction and genetics	2009	1058-0468	10.1007/s10815-009-9343-5
Cell Phones and Male Infertility: A Review of Recent Innovations in Technology and Consequences	Agarwal, Ashok; Singh, Aspinder; Hamada, Alaa; Kesari, Kavindra	International Braz J Urol	2011	1677-5538	10.1590/S1677-55382011000400002
Combined aerobic and resistance exercise training for improving reproductive function in infertile men: a randomized controlled trial	Maleki, Behzad Hajizadeh; Tartibian, Bakhtyar	Applied Physiology Nutrition and Metabolism	2017	1715-5312	10.1139/apnm-2017-0249
Effect of Coenzyme Q10 supplementation on antioxidant enzymes activity and oxidative stress of seminal plasma: a double-blind randomised clinical trial	Nadjarzadeh, A.; Shidfar, F.; Amirjannati, N.; Vafa, M. R.; Motevalian, S. A.; Gohari, M. R.; Kakhki, S. A. Nazeri; Akhondi, M. M.; Sadeghi, M. R.	Andrologia	2014	0303-4569	10.1111/and.12062
Effect of pentoxifylline on semen parameters, reproductive hormones, and seminal plasma antioxidant capacity in men with idiopathic infertility: a randomized double-blind placebo-controlled study	Safarinejad, Mohammad Reza	International urology and nephrology	2011	0301-1623	10.1007/s11255-010-9826-4
Increased oxidative stress and oxidative damage associated with chronic bacterial prostatitis	Zhou, J. F.; Xiao, W. Q.; Zheng, Y. C.; Dong, J.; Zhang, S. M.	Asian Journal of Andrology	2006	1008-682X	10.1111/j.1745-7262.2006.00144.x
Lifestyle and fertility: the influence of stress and quality of life on male fertility	Ilacqua, Alessandro; Izzo, Giulia; Emerenziani, Gian Pietro; Baldari, Carlo; Aversa, Antonio	Reproductive Biology and Endocrinology	2018	1477-7827	10.1186/s12958-018-0436-9
Mechanisms of male infertility: Role of antioxidants	Sheweita, S. A.; Tilmisany, A. M.; Al-Sawaf, H.	Current Drug Metabolism	2005	1389-2002	10.2174/138920005774330594
Melatonin and Male Reproductive Health: Relevance of Darkness and Antioxidant Properties	Rocha, C. S.; Rato, L.; Martins, A. D.; Alves, M. G.; Oliveira, P. F.	Current Molecular Medicine	2015	1566-5240	10.2174/1566524015666150505155530
Pro-oxidative and anti-oxidative imbalance in human semen and its relation with male fertility	Garrido, N.; Meseguer, M.; Simon, C.; Pellicer, A.; Remohi, J.	Asian Journal of Andrology	2004	1008-682X	
Resistance exercise modulates male factor infertility through anti-inflammatory and antioxidative mechanisms in infertile men: A RCT	Maleki, Behzad Hajizadeh; Tartibian, Bakhtyar	Life Sciences	2018	0024-3205	10.1016/j.lfs.2018.04.039
Role of reactive oxygen species in male infertility	Sharma, R. K.; Agarwal, A.	Urology	1996	0090-4295	10.1016/S0090-4295(96)00313-5
Role of Withania somnifera (Ashwagandha) in the management of male infertility	Sengupta, Pallav; Agarwal, Ashok; Pogrebetskaya, Maria; Roychoudhury, Shubhadeep; Durairajanayagam, Damayanthi; Henkel, Ralf	Reproductive Biomedicine Online	2018	1472-6483	10.1016/j.rbmo.2017.11.007

The Effects of Honey Supplementation on Seminal Plasma Cytokines, Oxidative Stress Biomarkers, and Antioxidants During 8 Weeks of Intensive Cycling Training	Tartibian, Bakhtyar; Maleki, Behzad Hajizadeh	Journal of andrology	2012	0196-3635	10.2164/jandrol.110.012815
The effects of three different exercise modalities on markers of male reproduction in healthy subjects: a randomized controlled trial	Maleki, Behzad Hajizadeh; Tartibian, Bakhtyar; Chehrazi, Mohammad	Reproduction	2017	1470-1626	10.1530/REP-16-0318
The hazardous effects of tobacco smoking on male fertility	Dai, Jing-Bo; Wang, Zhao-Xia; Qiao, Zhong-Dong	Asian Journal of Andrology	2015	1008-682X	10.4103/1008-682X.150847
Variations in Antioxidant Genes and Male Infertility.	Yu, Bolan; Huang, Zhaofeng	BioMed research international	2015		10.1155/2015/513196
